# Evaluation of the nutritive value of muiumba (*Baikiaea plurijuga*) seeds: chemical composition, *in vitro* organic matter digestibility and *in vitro* gas production

**DOI:** 10.1186/2193-1801-3-311

**Published:** 2014-06-25

**Authors:** Miguel A M Rodrigues, Ana L Lourenço, John W Cone, Fernando M Nunes, Ana S Santos, José M M Cordeiro, Cristina M V Guedes, Luis M M Ferreira

**Affiliations:** Department of Animal Science, Animal Science and Veterinary Research Centre, Universidade de Trás-os-Montes e Alto Douro, P.O. Box 1013, 5001-801 Vila Real, Portugal; Department of Chemistry, Chemistry Research Centre, Universidade de Trás-os-Montes e Alto Douro, Vila-Real, Portugal; Wageningen Institute of Animal Sciences, Animal Nutrition Group, Wageningen University, Wageningen, The Netherlands; Department of Veterinary Medicine, Animal Science and Veterinary Research Centre, Universidade Vasco da Gama, Coimbra, Portugal; Department of Animal Production, Faculty of Veterinary Medicine, Universidade José Eduardo dos Santos, Huambo, Angola

**Keywords:** Muiumba seeds, Nutritive value

## Abstract

One of the main constraints hindering the increase of animal production in semi-arid regions of Africa is the inadequate supply of nutrients during the dry season. Incorporation of alternative feed resources in ruminant diets during this period could be a viable approach to overcome these limitations. The objective of this study was to evaluate the nutritive value of muiumba (*Baikiaea plurijuga*) tree seeds as an alternative nutrient source for ruminants. Muiumba seeds were compared to other eight feedstuffs including two cereal grains (corn and oat), two wheat by-products (wheat bran and distilled wheat) and four protein meals (coconut meal, sunflower meal, soybean meal and rapeseed meal) as to its chemical composition, *in vitro* organic matter digestibility (IVOMD) and *in vitro* gas production. The moderate crude protein concentrations (145 g/kg DM) of muiumba seeds indicate that this feedstuff could not be used as a protein supplement, contrarily to the majority of multipurpose tree seeds. Although the starch content was scarce (15 g/kg DM), the low neutral detergent fibre (235 g/kg DM), low molecular weight sugar (76.1 g/kg DM) and non-starch polysaccharide (510.5 g/kg DM) contents indicate that this feedstuff has potential feeding value. This was confirmed by the IVOMD (0.770) and by the data provided by the *in vitro* gas production showing that muiumba seeds had high (*P* < 0.05) maximum gas production and fractional fermentation rates, suggesting that these seeds are characterized by a highly fermentable fraction.

## Introduction

The nutritive value and production cycle of natural pastures depend on prevailing climate and soil conditions. Ruminant production in semiarid regions, especially small ruminants, is mainly extensive and highly dependent on rangeland resources including crop residues and their by-products (Ben Salem and Smith 2008). These feeds are characterized by high levels of fibre and low levels of energy, protein, minerals and vitamins, promoting low levels of voluntary intake and digestibility, which leads to an intake below the animal’s requirements during long periods of the year (Sarwatt et al. 2002). In fact, nutritional deficiencies are accepted to be one of the major technical limitations to increase productivity of ruminant livestock in the tropics (Camero et al. 2001). Several alternatives have been proposed to overcome feed limitations in such extensive production systems. Among these, shrubs and fodder trees (leaves and seeds) are considered to have high potential value as source of essential nutrients. This is due to their high nutritive value, in terms of protein and mineral content, but also to its tolerance to a wide range of management practices, high longevity and capacity to produce fodder when other species become dormant to avoid adverse climatic conditions (Paterson et al. 1998; Camero et al. 2001; Sarwatt et al. 2002; Ben Salem et al. 2004).

Although many studies have been conducted to evaluate the nutritive value of multipurpose trees and shrub legumes, results from tree seeds are scarce on literature. The existing studies show that these seeds can be an interesting nutrient source (Salawu et al. 1999; Smith et al. 2005; Mlambo et al. 2008). Native trees such as muiumba (*Baikiaea plurijuga*) are important sources of browse for ruminant production within the areas of Miombo woodlands of Africa, namely in the Cunene region. Small ruminants produced in this region exhibit, in particular during the dry season (from May to August), a natural tendency to ingest the young leaves and seeds of *Baikiaea plurijuga* as a complement to their limited availability and low nutritive herbaceous diet. In spite of this well-known behaviour, the nutritive value of these seeds cannot be found in the literature. The objective of this study was to evaluate the nutritive value of muiumba seeds and to compare it with conventional ruminant feedstuffs to verify its potential as an alternative feed in ruminant extensive production systems.

## Materials and methods

### Feedstuffs

Nine feedstuffs were evaluated in this study. Two cereal grains (corn and oat), two wheat by-products (wheat bran and distilled wheat), four protein meals (coconut meal, sunflower meal, soybean meal and rapeseed meal) and muiumba seeds were analysed. Feeds were chosen in order to cover a wide range in chemical composition and *in vitro* digestibility. Mature muiumba seeds were collected in the semiarid region of the Cunene basin (Angola) during the dry season. Samples were dried in a forced-air oven (Venticell, Munchen, Germany) at 60°C for 24 h for dry matter (DM) determination. Dried samples were ground to pass 1 mm screen (Retsch, Cutting mill, model SM1, Haan, Germany) and stored in airtight flasks at room temperature for subsequent laboratorial analysis. For starch and non-starch polysaccharides (NSP) analysis, samples were ground to pass a 0.5 mm screen (Cyclotec, Tecator, Hoganas Sweden).

### Chemical analysis

Samples were analysed in quadruplicate using the procedures described by AOAC, Association of Official Analytical Chemists (1990) for ash (#942.05), crude protein (#954.01) and ether extract (#920.39). Neutral detergent fibre (NDF) was determined by the detergent procedures of Van Soest et al. (1991). Sodium sulphite was not added and heat stable amylase (Sigma A3306, St. Louis, Ill, USA) was only added to cereal grains and wheat by-products samples. Klason lignin (KL) was determined using a two stage sulphuric acid hydrolysis according to the method proposed by Theander and Westerlund (1986).

Low molecular weight sugars (LMW) were analysed according to the methods of Knudsen (1997) with minor modifications. Samples of approximately 500 mg were weighed into 50 mL centrifuge tubes. Fifteen mL of an ethanol solution (500 mL/L), including 2 mL of an internal standard (arabinose, 1 mg/mL), were added and the samples sonicated and extracted in a water bath for 60 min at 65°C. During extraction the tubes were mixed (vortex mixer) three times and finally centrifuged (2200 g, 20 min). An aliquot of 1 mL was removed from the supernatant and diluted with 5 mL of water and analysed by anion-exchange chromatography with pulsed amperometric detection (HPAEC-PAD). The HPAEC system used consisted of the model ICS-5000 (Thermo Fisher Scientific Inc., Dionex, USA) equipped with an ED40 electrochemical detector (Thermo Fisher Scientific Inc., Dionex, USA) with a working gold electrode. Low molecular weight sugars were analysed by isocratic elution with 0.05 M NaOH containing 0.002 M of Ba(OH)_2_ at 0.5 mL/min with a CarboPac PA-20 column (Thermo Fisher Scientific Inc., Dionex, USA). Sugar composition from the total and insoluble non-starch polysaccharides (NSP) were analysed by isocratic elution with 0.018 M NaOH containing 0.002 M of Ba(OH)_2_ at 1 mL/min with a CarboPac PA-1 column (Thermo Fisher Scientific Inc., Dionex, USA). The injection volume was 10 μL, and the column and detector temperature was set at 25°C. All individual sugar values were converted to their equivalent polysaccharide value using appropriate conversion factors; 0.88 for pentoses and deoxyhexoses and 0.9 for other hexoses.

The starch content was determined according to the enzymatic method proposed by Salomonsson et al. (1984), with amyloglucosidase from *Aspergillus niger* (Sigma, A3042; 6000 U/ml) and heat stable amylase (Sigma A3306, Ill) using a spectrometer (U2000, Hitachi Ltd., Japan).

Total, soluble, and insoluble NSP were determined using the Knudsen (1997) procedures with minor modifications. Briefly, samples were weighed into 50 mL centrifuge tubes. Acetate-buffer with CaCl_2_ (0.1 M; 0.02 M; pH 5.0; 9.8 ml), termostable α-amylase (Termamyl, Novo Nordisk A/S.; 100 μL) was added and the samples were incubated for 1 h at 100°C. Complete degradation of starch was achieved after 2 h with β-glucanase free amyloglucosidase from *Aspergillus niger* (Boehringer Mannheim GmbH, Cat No. 1202 367:135 U/ml; 100 μL) at 60°C. Soluble NSP were precipitated with 4 volumes of an ethanol solution (990 mL/L) for 1 h on ice bath, the tubes centrifuged (4600 rpm; 10 min) and the supernatant discarded. Residues were washed twice with an ethanol solution (850 mL/L) and once with acetone and dried in a hood overnight. Polysaccharides in starch free residues were treated with 12 M H_2_SO_4_ (35°C 60 min) and hydrolysed to monosaccharides with 1 M (100°C. 120 min). An internal standard (2-deoxyglucose 1 mg/mL; 500 μL) was added to the hydrolysate aliquot. Neutral sugar analysis was performed by HPAEC-PAD (section 2.2.1.). The uronic acids were measured spectrophotometrically with D-galacturonic acid solutions as standards according to the method described by Scott (1979).

The soluble NSP in the starch-free residue were extracted by a phosphate buffer (Englyst et al. 1982) at neutral pH (0.2 M; 100°C; 60 min; pH 7.0) and the neutral and acidic sugars in the insoluble NSP were analysed as described for total NSP.

### Incubation procedures

Two cows, weighing 500 ± 36 kg live weight and fitted with permanent ruminal cannulae (Bar Diamond Inc., Parma ID, USA), were used to collect rumen fluid. Cows were housed in individual stalls in a ventilated barn. The diet consisted of meadow hay and commercial concentrate making 75:25 ratio on a DM basis and was offered at 1.2 times the maintenance requirements (Thomas, 2004). Animals were fed in equal portions at 0800 and 1600 h and had free access to water and mineral-vitamin blocks. The concentrate was offered to the animals prior to the hay. Rumen fluid samples were drawn 2 h after the morning feed into pre-warmed insulated flasks previously filled with CO_2_. Rumen fluid was strained through 4 layers of cheesecloth and kept at 39°C under CO_2_.

*In vitro* organic matter digestibility (IVOMD) was determined according to Tilley and Terry (1963) modified by Marten and Barnes (1980). Each sample was incubated in quadruplicate in one series. Fermentations were in 50 mL centrifuge tubes containing 250 mg of sample and 25 ml of buffered rumen fluid solution. Immediately after addition of buffer solution, flasks were closed with rubber stoppers fitted to a Bunsen valve. After 48 h of incubation a pepsin/HCl solution was added and the incubation was prolonged by 24 h.

Rumen fluid used to measure *in vitro* gas production was mixed (1:2 v/v) with an anaerobic buffer/mineral solution (Cone et al. 1996). All laboratory handling was under continuous flushing with CO_2_. Samples (500 mg) were accurately weighed into 250 mL serum bottles (Schott, Mainz, Germany) and incubated in 60 ml buffered rumen fluid. Each sample was incubated in two series, completed on different days. Gas production was recorded every 20 min for 72 h using a fully automated system (Cone et al. 1996).

### Calculations and statistical analysis

Gas curves were fitted by iteration to a mono-phasic model as described by Groot et al. (1996) as:


where: *A* = estimated asymptotic gas production; *B* = time of incubation at which half of the asymptotic gas production has been formed; *C* = sharpness of the switching characteristic for the profile.

Maximum rate of gas production (*R*_*max*_*G*) and the time at which maximum rate of gas production is reached (*TR*_*max*_*G*) were calculated according to Yang et al. (2005) as:


and


Fractional rate of substrate fermentation (*R*) was calculated using the equation of Groot et al. (1996) as:


All data were expressed in terms of ml of gas/g OM fermented.

*In vitro* organic matter digestibility and *in vitro* gas production parameters were analysed according to a completely randomized design using GLM of SAS (SAS, 2004). Differences between treatment means were determined using Tukey’s test with a predetermined significance level of *P* < 0.05.

## Results

The chemical composition of the feeds is presented in Table 1. Muiumba seeds were characterized by low levels of protein (145 g/kg DM) compared to protein concentrates normally used in ruminant feeding namely rapeseed and soybean meals. Its NDF content was rather low (235 g/kg DM) compared to the majority of the other feeds, with the exception of corn and soybean meal. Nevertheless, lignin content was quite high (180 g/kg DM) ranking above all the other feeds. Muiumba seeds also presented low starch concentrations (15 g/kg DM).

**Table 1:** **Chemical composition of feedstuffs (g/kg DM) compared in the present study, results are expressed as mean ± SE**

Feeds	Ash	CP	NDF	KL	EE	Starch
Muiumba seeds	26 ± 0.3	145 ± 3.2	235 ± 7.9	180 ± 10.5	101 ± 4.7	15 ± 1.2
Corn	15 ± 0.7	87 ± 3.1	100 ± 8.3	34 ± 3.8	46 ± 5.6	789 ± 20.4
Oats	25 ± 0.7	100 ± 4.2	315 ± 7.7	104 ± 9.8	51 ± 4.9	464 ± 35.5
Distilled wheat	83 ± 1.3	329 ± 7.4	453 ± 12.9	87 ± 6.9	47 ± 5.1	25 ± 2.7
Wheat bran	94 ± 1.8	113 ± 1.6	547 ± 7.6	148 ± 9.9	41 ± 4.3	154 ± 14.3
Coconut meal	71 ± 1.6	213 ± 7.2	555 ± 10.2	66 ± 4.4	56 ± 2.0	25 ± 2.6
Sunflower meal	65 ± 0.3	257 ± 4.4	485 ± 8.8	129 ± 7.9	24 ± 3.0	15 ± 0.7
Rapeseed meal	73 ± 0.3	353 ± 5.6	325 ± 11.3	139 ± 4.4	38 ± 1.5	25 ± 1.3
Soybean meal	70 ± 0.9	444 ± 8.7	147 ± 3.1	36 ± 4.1	19 ± 2.1	27 ± 0.7

*CP* crude protein, *NDF* neutral detergent fibre, *KL* Klason lignin, *EE* ether extract.

As to the composition in LMW sugars (Table 2) muiumba seeds ranked (76.1 g/kg DM) along the feeds presenting medium concentrations. The relative proportion of the individual LMW sugars varied among feeds. Muiumba seeds mainly contain maltose, raffinose and sucrose being the raffinose content particularly high (25.0 g/kg DM) compared to the other feedstuffs.

**Table 2:** **Low molecular weight sugars of feedstuffs (g/kg DM) compared in the present study, results are expressed as mean ± SE**

Feeds	Glucose	Frutose	Sucrose	Maltose	Verbascose	Raffinose	Total
Muiumba seeds	0.18 ± 0.01	4.24 ± 0.12	13.83 ± 0.55	32.88 ± 1.97	ND	25.00 ± 1.41	76.1
Corn	1.49 ± 0.05	1.77 ± 0.05	18.05 ± 0.41	17.62 ± 0.77	ND	1.82 ± 0.09	40.8
Oats	0.52 ± 0.03	0.76 ± 0.02	8.03 ± 0.25	7.55 ± 1.97	0.50 ± 0.02	8.46 ± 0.51	28.8
Distilled wheat	0.51 ± 0.02	ND	6.07 ± 0.36	1.04 ± 0.39	0.19 ± 0.01	1.19 ± 0.07	9.3
Wheat bran	2.48 ± 0.10	1.61 ± 0.06	20.60 ± 1.23	49.60 ± 3.27	0.67 ± 0.04	12.04 ± 0.47	87.0
Coconut meal	0.43 ± 0.02	5.30 ± 0.11	52.66 ± 2.64	3.66 ± 0.07	ND	5.06 ± 0.27	67.1
Sunflower meal	2.96 ± 0.11	5.09 ± 0.13	39.60 ± 2.08	203.30 ± 10.9	1.16 ± 0.07	0.78 ± 0.03	252.6
Rapeseed meal	0.78 ± 0.04	1.26 ± 0.04	58.59 ± 3.52	1.00 ± 0.04	0.99 ± 0.03	1.78 ± 0.10	62.7
Soybean meal	0.11 ± 0.01	0.23 ± 0.01	39.75 ± 1.65	115.10 ± 6.46	28.90 ± 1.77	ND	184.2

*ND* not detected.

The composition of the NSP fraction is presented in Table 3 and differed greatly between feeds. Muiumba seeds, wheat bran, coconut and sunflower meals presented much higher NSP concentrations (around 500 g/kg DM) than cereal grains, distilled wheat, rapeseed and soybean meal. Muiumba seeds were characterized by an insoluble fraction that was twice the soluble one, glucose being the main component of NSP in muiumba seeds (59% of total NSP) - distributed in equal absolute amounts in soluble and insoluble fraction - and followed by xylose (19% of total NSP) and galactose (17% of total NSP).

**Table 3:** **Composition of non-starch polysaccharides (NSP) of feedstuffs (g/kg DM) compared in the present study, results are expressed as mean ± SE**

NSP	Muiumba seeds	Corn	Oats	Distilled wheat	Wheat bran	Coconut meal	Sunflower meal	Rapeseed meal	Soybean meal
Soluble NSP									
Fucose	0.2 ± 0.1	ND	ND	ND	ND	ND	ND	ND	ND
Arabinose	1.7 ± 0.2	0.6 ± 0.1	2.8 ± 0.2	25.5 ± 1.8	1.2 ± 0.1	0.2 ± 0.1	16.2 ± 1.3	14.5 ± 0.9	27.6 ± 2.3
Rhamnose	ND	ND	ND	ND	ND	ND	ND	ND	ND
Galactose	14.3 ± 1.1	0.2 ± 0.1	0.5 ± 0.1	1.0 ± 1.0	0.7 ± 0.1	1.1 ± 0.1	3.9 ± 0.4	0.4 ± 0.1	50.4 ± 4.0
Glucose	145.1 ± 12.9	30.2 ± 2.1	18.4 ± 1.2	54.5 ± 4.8	162.1 ± 14.8	10.1 ± 0.5	183.3 ± 10.9	22.8 ± 2.0	97.3 ± 8.7
Xylose	19.4 ± 2.4	30.3 ± 2.7	43.4 ± 3.9	27.2 ± 1.8	34.8 ± 2.5	2.4 ± 0.2	45.1 ± 3.1	5.6 ± 0.5	8.8 ± 0.8
Mannose	ND	11.5 ± 0.6	2.0 ± 0.2	0.3 ± 0.1	0.8 ± 0.1	113.1 ± 9.6	2.7 ± 0.3	0.8 ± 0.1	5.9 ± 0.6
Uronic acids	1.7 ± 0.2	1.0 ± 0.1	0.1 ± 0.1	0.4 ± 0.1	1.5 ± 0.1	0.5 ± 0.1	9.2 ± 0.8	0.9 ± 0.1	2.3 ± 0.2
Total	182.4 ± 13.1	73.8 ± 3.5	67.2 ± 4.1	108.9 ± 5.5	201.1 ± 15.0	127.4 ± 9.6	260.6 ± 11.4	45.0 ± 2.26	192.3 ± 9.9
Insoluble NSP									
Fucose	0.3 ± 0.1	ND	ND	ND	ND	ND	ND	ND	ND
Arabinose	33.3 ± 3.9	18.0 ± 1.1	7.6 ± 0.6	18.0 ± 1.5	55.0 ± 4.2	6.6 ± 0.6	16.9 ± 1.3	41.3 ± 0.4	8.0 ± 0.8
Rhamnose	ND	ND	ND	ND	ND	ND	ND	ND	ND
Galactose	72.2 ± 5.4	3.3 ± 0.2	2.7 ± 0.2	3.5 ± 0.3	5.3 ± 0.4	13.3 ± 1.2	5.3 ± 0.4	13.4 ± 1.0	12.5 ± 0.9
Glucose	150.0 ± 13.4	30.8 ± 1.6	66.4 ± 5.6	61.0 ± 5.1	217.8 ± 16.8	54.0 ± 4.3	135.6 ± 11.2	98.9 ± 8.1	25.8 ± 2.1
Xylose	78.6 ± 4.6	35.2 ± 2.5	77.7 ± 6.9	65.5 ± 5.8	239.3 ± 16.2	1.9 ± 0.2	80.8 ± 7.1	17.1 ± 1.6	5.8 ± 0.5
Mannose	ND	1.3 ± 0.1	1.5 ± 0.1	13.5 ± 1.1	1.9 ± 0.2	300.1 ± 25.2	7.0 ± 0.6	5.3 ± 0.5	2.2 ± 0.2
Uronic acids	5.3 ± 0.6	2.6 ± 0.3	2.6 ± 0.2	3.2 ± 0.3	5.1 ± 0.4	4.7 ± 0.4	18.6 ± 1.7	23.2 ± 1.9	10.4 ± 1.0
Total	339.7 ± 15.7	91.2 ± 3..3	158.5 ± 8.9	164.7 ± 8.0	524.4 ± 23.6	380.6 ± 25.6	264.1 ± 13.5	199.2 ± 8.6	64.7 ± 2.7
Total NSP	522.1 ± 20.4	165.0 ± 4.8	225.7 ± 9.8	273.6 ± 9.7	725.5 ± 27.9	508.0 ± 27.3	524.7 ± 17.7	244.2 ± 8.8	257.0 ± 10.3

*ND* not detected.

The IVOMD differed (*P* < 0.001) between feeds (Table 4), being that of muiumba seeds relatively high (0.770). Gas production profiles showed variations (*P* < 0.001) in both rate and extent of fermentation among feedstuffs (Table 4). The estimated asymptotic gas production of muiumba seeds showed an intermediate value (257.6 ml/g OM, *P* < 0.05) which was similar to the values found for oats, distilled wheat and coconut, rapeseed and soybean meal. The maximum rate of gas production (*RmaxG*) and the fractional rate of fermentation (*R*) showed highest values (*P* < 0.05) for oats, muiumba seeds and corn (23.8, 24.9, and 26.7 mL/h, and 0.107, 0.130 and 0.100/h, respectively).

**Table 4:** **Parameters of gas production profiles fitted with mono-phasic Groot’s model and** ***in vitro*** **digestibility of feedstuffs compared in the present study**

Feeds	Gas production parameters ^a^						IVOMD ^b^
	*A (ml/g OM)*	*B (h)*	*C*	*RmaxG (ml/h)*	*TRmaxG (h)*	*R (/h)*	
Muiumba seeds	257.6^bc^	6.8^a^	2.07^c^	24.9^ef^	4.1^cd^	0.130^e^	0.770^d^
Corn	356.6^d^	8.8^cd^	2.03^c^	26.7^f^	5.1^d^	0.100^d^	0.851^g^
Oats	271.2^bc^	6.9^ab^	1.55^b^	23.8^e^	2.6^ab^	0.107^d^	0.709^c^
Distilled wheat	262.6^bc^	9.9^de^	1.36^ab^	16.7^bc^	2.5^ab^	0.074^b^	0.873^h^
Wheat bran	200.4^a^	13.0^f^	1.21^a^	10.4^a^	1.8^a^	0.057^a^	0.575^a^
Coconut meal	287.6^c^	12.5^f^	1.93^c^	14.8^b^	6.9^e^	0.068^b^	0.807^e^
Sunflower meal	195.6^a^	6.9^ab^	1.48^b^	17.2^cd^	2.3^ab^	0.105^d^	0.678^b^
Rapeseed meal	256.7^b^	8.1^bc^	1.48^b^	19.4^d^	2.7^ab^	0.090^c^	0.825^f^
Soybean meal	282.0^bc^	10.4^e^	1.48^b^	16.6^bc^	3.4^bc^	0.070^b^	0.968^i^
SEM	9.62	0.39	0.073	0.70	0.39	0.003	0.005
Probability	<0.001	<0.001	<0.001	<0.001	<0.001	<0.001	<0.001

Means in rows with different letters differ at *P* < 0.05.

^a^ *A* is the estimated asymptotic gas production; *B* is the time of incubation at which half of the asymptotic gas production has been formed; *C* is the sharpness of the switching characteristic for the profile; *RmaxG* is the maximum rate of gas production; *TRmaxG* is the time at which maximum rate of gas production is reached; *R* is the fractional rate of fermentation.

^b^ *In vitro* organic matter digestibility.

## Discussion

The chemical composition of muiumba seeds showed that this feedstuff could only supply moderate concentrations of protein making unviable its utilization as a protein supplement. Generally, it is assumed that a feed above 200 g/kg DM crude protein and less than 180 g/kg DM crude fibre (Harris et al. 1968, 1980) could be considered a protein supplement. The low protein value for muiumba may come as a surprise since it is a legume tree and thus a higher protein value was expected. In fact, although tree seeds can be used as protein sources, even for humans (Adewusi et al. 2003), CP values reported herein are lower than those reported for seeds from other types of fodder trees. Data concerning seeds from different species of Acacia trees show an average CP content around 300 g/kg DM (Mahala and Elseed 2007). Other multipurpose trees with a wide agricultural use in Africa, including human consumption and animal feed, are Moringa species. Results presented by Melesse et al. (2009) for seeds of *Moringa stenopetala* show higher CP values, around 400 g/kg DM. Generalizations maybe somewhat misleading as chemical composition is subjected to wide fluctuations depending on soil and climate characteristics. A recent study from Soliva et al. (2008) confirms the importance of multipurpose tree seeds as protein sources, showing that fruits and seeds medium CP concentration of ten different plants was around 300 g/kg DM.

Although its starch content is practically inexistent, the reduced NDF concentration together with the relatively high content in LMW sugars and NSP indicate that muiumba seeds could have potential feeding value as a dietary component for ruminants in areas where one of the most important factors limiting productivity is limited feed supply. The high glucose content as well as the xylose and galactose concentration of NSP (Table 3) suggests that muiumba seeds are rich in glucans, xyloglucans and galactans. This is not surprising since legume seeds are usually rich in these compounds (Buckeridge et al. 1995; 2000; Srivastava and Kapoor 2005). Although xyloglucan, one of the most abundant hemicelluloses, can generally be found in the plant cell wall structure it can also be a storage polysaccharide in some tree seeds. Like starch, galactan-containing polysaccharides are also considered to be storage carbohydrates and thus easily fermentable by the rumen microbial population. Furthermore, as lignin concentration is very similar to the NDF content, the majority of the fibrous fraction of the muiumba seeds is basically lignin and a small amount of cellulose. These concentrations mainly derive from the hulk, which in these seeds is firmly coated making its removal quite complicated. In this way, it could be argued that the fibrous content of muiumba seeds would not affect the digestibility of other compounds. This is confirmed by the values of IVOMD presented in Table 4. In fact, assuming that the NDF content of muiumba seeds (235 g/kg DM), mainly present in the hulk, is totally indigestible then the remaining fraction is practically totally digestible, as the IVOMD of muiumba averaged 0.770. This feature would rank muiumba seeds along the feedstuffs (Table 4) with higher digestibility in case the hulk could be removed.

Data for most of the seeds from other types of fodder trees also show that one of the main constraints on its utilization as animal feed is the inhibitory effect of lignin, limiting intake and digestibility of nutrients, as well as the concentrations of other anti-nutritional compounds. As previously discussed, this is not the case for muiumba seeds. Additionally, although values reported in the literature are quite variable within fodder tree species, muiumba seeds presented higher IVOMD when compared to the results reported by Melesse et al. (2009) for organic matter digestibility of *Moringa stenopetala* (523 g/kg OM) and Salawu et al. (1999) for *in vitro* DM degradation of *Calliandra calothyrsus* (471 g/kg DM) seeds. Data presented by Aganga and Mosase (2001) also point out to low dry matter digestibility values for the seeds of indigenous trees species of southern Africa, with a value around 400 g/kg DM. Tree seeds from the reported species generally present quite variable digestibility values. This feature is expected since it depends on its chemical composition. The phenolic content of fodder trees and shrubs is usually considered as one of the main factors that negatively affects its digestibility, and several authors have reported this fact (; Smith et al. 2005; Ben Salem and Smith 2008; Blache et al. 2008). A large number of non-conventional feedstuffs, such as tree seeds, may contain secondary compounds that are potentially toxic, due to their structure and rate of intake (Blache et al. 2008). Whether muiumba is rich on phenolic compounds will have to be evaluated in future trials. Nevertheless, the IVOMD value found in our study indicates that whatever the concentration of phenolic compounds in muiumba seeds its nutritive value is high.

**Figure 1:**
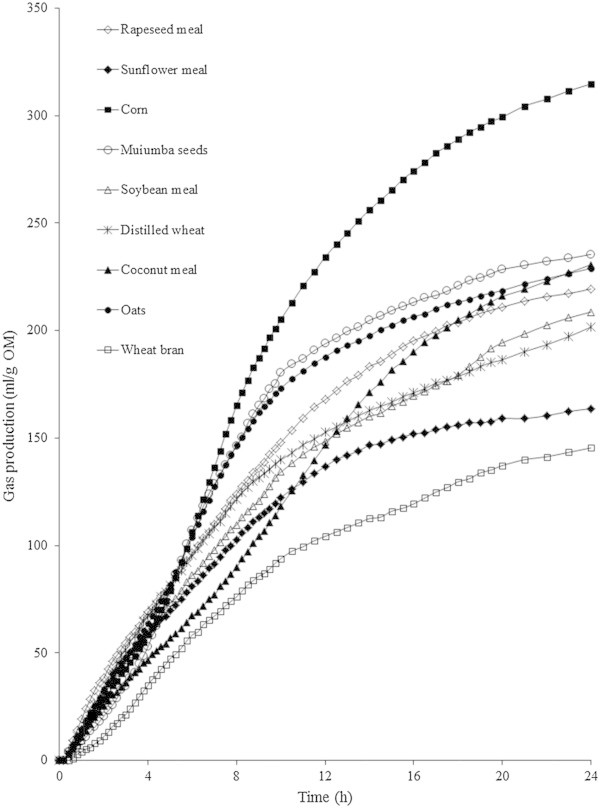
**Gas production curves of feedstuffs compared in the present study.**

The *in vitro* gas production data are in agreement with the IVOMD results, and confirm the muiumba seeds potential as an animal feed. The estimated asymptotic gas production (*A*) ranked it as similar to other commonly used supplements. Furthermore, its maximum gas production rate (RmaxG) and its fractional fermentation rate (R) showed that muiumba seeds are characterized by a highly fermentable fraction. By applying the equation suggested by Menke and Steingass (1988), for metabolizable energy calculations, Muimba seeds presented values of 9.1 MJ/kg DM. This feature mainly reflects the close association between the chemical composition on low molecular weight sugars and non-starch polysaccharides and fermentation rates, meaning that both soluble and the majority of non-soluble components have high rumen fermentability. The description of gas production profiles can be attributed to the fermentation of several fractions (Cone et al. 1997); a sub-curve outlining the water-soluble fraction, another outlining the non-soluble fraction and an additional one describing the microbial turnover. In a study comparing 20 feed samples Van Gelder et al. (2005) verified that gas production of the soluble and insoluble fractions could be estimated from the gas produced by each substrate at 3 h and 20 h incubation, respectively. These data support the findings of Cone et al. (1997) that for different types of substrate also pointed out that the gas production sub-curves could be estimated using this approach. In this way, sugars and the majority of proteins are considered to ferment within the first 3 hours of fermentation while starch and NSP are fermented between 3 and 20 hours of fermentation, depending on the substrates chemical composition. In fact, fibrous feedstuffs will tend to show lower fermentation rates and the period of fermentation of the non-soluble fraction will be more extensive due to the higher concentration of cell wall components. The gas production observed for the different feedstuffs used in this study (Figure 1) indicate that after 5 h of fermentation muiumba seeds gas production had a significant contribution of the non-soluble fraction, confirming that the NSP fraction is highly fermentable.

## Conclusions

This is the first study presenting data on the nutritional value of muiumba seeds. Our results suggest that although muiumba seeds have a moderate concentration of crude protein and vestigial starch contents, its high digestibility and fermentation patterns suggest that its carbohydrate fraction is easily fermented. Nevertheless, further studies should be performed in order to evaluate the presence of anti-nutritional constituents such as tannins and other phenolic compounds.

## Abbreviations

A: Estimated asymptotic gas production
B: Time of incubation at which half of the asymptotic gas production (A) has been formed
C: Sharpness of the switching characteristic for the gas production profile
CP: Crude protein
DM: Dry matter
EE: Ether extract
IVOMD: *In vitro* OM digestibility
KL: Klason lignin
LMW: Low molecular weight
NDF: Neutral detergent fibre
OM: Organic matter
NSP: Non-starch polysaccharides
R: Fractional rate of substrate fermentation
RmaxG: Maximum rate of gas production
TRmaxG: Time at which maximum rate of gas production is reached.
